# Socioeconomic Factors and All Cause and Cause-Specific Mortality among Older People in Latin America, India, and China: A Population-Based Cohort Study

**DOI:** 10.1371/journal.pmed.1001179

**Published:** 2012-02-28

**Authors:** Cleusa P. Ferri, Daisy Acosta, Mariella Guerra, Yueqin Huang, Juan J. Llibre-Rodriguez, Aquiles Salas, Ana Luisa Sosa, Joseph D. Williams, Ciro Gaona, Zhaorui Liu, Lisseth Noriega-Fernandez, A. T. Jotheeswaran, Martin J. Prince

**Affiliations:** 1 King's College London Institute of Psychiatry, Section of Epidemiology, Health Service and Population Research Department, London, United Kingdom; 2 Internal Medicine Department, Universidad Nacional Pedro Henriquez Ureña, Santo Domingo, Dominican Republic; 3 Department of Psychiatry, Universidad Peruana Cayetano Heredia, Lima, Peru; 4 Peking University, Institute of Mental Health, Beijing, China; 5 Medical University of Havana, Havana, Cuba; 6 Medicine Department, Caracas University Hospital, Caracas, Venezuela; 7 Faculty of Medicine, Universidad Central de Venezuela, Caracas, Venezuela; 8 National Institute of Neurology and Neurosurgery of Mexico, Autonomous National University of Mexico, Mexico City, Mexico; 9 Community Health Department, Voluntary Health Services, Chennai, India; 10 Clinica Loira, Caracas, Venezuela; 11 Mental Health Community Centre of Marianao, Havana, Cuba; 12 Indian Institute of Public Health, Hyderabad, India; Umeå Centre for Global Health Research, Sweden

## Abstract

**Background:**

Even in low and middle income countries most deaths occur in older adults. In Europe, the effects of better education and home ownership upon mortality seem to persist into old age, but these effects may not generalise to LMICs. Reliable data on causes and determinants of mortality are lacking.

**Methods and Findings:**

The vital status of 12,373 people aged 65 y and over was determined 3–5 y after baseline survey in sites in Latin America, India, and China. We report crude and standardised mortality rates, standardized mortality ratios comparing mortality experience with that in the United States, and estimated associations with socioeconomic factors using Cox's proportional hazards regression. Cause-specific mortality fractions were estimated using the InterVA algorithm. Crude mortality rates varied from 27.3 to 70.0 per 1,000 person-years, a 3-fold variation persisting after standardisation for demographic and economic factors. Compared with the US, mortality was much higher in urban India and rural China, much lower in Peru, Venezuela, and urban Mexico, and similar in other sites. Mortality rates were higher among men, and increased with age. Adjusting for these effects, it was found that education, occupational attainment, assets, and pension receipt were all inversely associated with mortality, and food insecurity positively associated. Mutually adjusted, only education remained protective (pooled hazard ratio 0.93, 95% CI 0.89–0.98). Most deaths occurred at home, but, except in India, most individuals received medical attention during their final illness. Chronic diseases were the main causes of death, together with tuberculosis and liver disease, with stroke the leading cause in nearly all sites.

**Conclusions:**

Education seems to have an important latent effect on mortality into late life. However, compositional differences in socioeconomic position do not explain differences in mortality between sites. Social protection for older people, and the effectiveness of health systems in preventing and treating chronic disease, may be as important as economic and human development.

*Please see later in the article for the Editors' Summary*

## Abstract 2

Cleusa Ferri and colleagues studied mortality rates in over 12,000 people aged 65 years and over in Latin America, India, and China and showed that chronic diseases are the main causes of death and that education has an important effect on mortality.

## Editors' Summary

**Background:**

Worldwide, half of all deaths occur in people aged 60 or older. Yet mortality among older people is a neglected topic in global health. In high income countries, where 84% of people do not die until they are aged 65 years or older, the causes of death among older people and the factors (determinants) that affect their risk of dying are well documented. In Europe, for example, the leading causes of death among older people are heart disease, stroke, and other chronic (long-term) diseases. Moreover, as in younger age groups, having a better education and owning a house reduces the risk of death among older people. By contrast, in low and middle income countries (LMICs), where three-quarters of deaths of older people occur, reliable data on the causes and determinants of death among older people are lacking, in part because many LMICs have inadequate vital registration systems—official records of all births and deaths.

**Why Was This Study Done?:**

In many LMICs, chronic diseases are replacing communicable (infectious) diseases as the leading causes of death and disability—health experts call this the epidemiological transition (epidemiology is the study of the distribution and causes of disease in populations)—and the average age of the population is increasing (the demographic transition). Faced with these changes, which occur when countries move from a pre-industrial to an industrial economy, policy makers in LMICs need to introduce measures to improve health and reduce deaths among older people. However, to do this, they need reliable data on the causes and determinants of death in this section of the population. In this longitudinal population-based cohort study (a type of study that follows a group of people from a defined population over time), researchers from the 10/66 Dementia Research Group, which is carrying out population-based research on dementia, aging, and non-communicable diseases in LMICs, investigate the patterns of mortality among older people living in Latin America, India, and China.

**What Did the Researchers Do and Find?:**

Between 2003 and 2005, the researchers completed a baseline survey of people aged 65 years or older living in six Latin American LMICs, China, and India. Three to five years later, they determined the vital status of 12,373 of the study participants (that is, they determined whether the individual was alive or dead) and interviewed a key informant (usually a relative) about each death using a standardized “verbal autopsy” questionnaire that includes questions about date and place of death, and about medical help-seeking and signs and symptoms noted during the final illness. Finally, they used a tool called the InterVA algorithm to calculate the most likely causes of death from the verbal autopsies. Crude mortality rates varied from 27.3 per 1,000 person-years in urban Peru to 70.0 per 1,000 person-years in urban India, a three-fold difference in mortality rates that persisted even after allowing for differences in age, sex, education, occupational attainment, and number of assets among the study sites. Compared to the US, mortality rates were much higher in urban India and rural China; much lower in urban and rural Peru, Venezuela, and urban Mexico; but similar elsewhere. Although several socioeconomic factors were associated with mortality, only a higher education status provided consistent independent protection against death in statistical analyses. Finally, chronic diseases were the main causes of death; stroke was the leading cause of death at all the sites except those in rural Peru and Mexico.

**What Do These Findings Mean?:**

These findings identify the main causes of death among older adults in a range of LMICs and suggest that there is an association of education with mortality that extends into later life. However, these findings may not be generalizable to other LMICs or even to other sites in the LMICs studied, and because some of the information provided by key informants may have been affected by recall error, the accuracy of the findings may be limited. Nevertheless, these findings suggest how health and mortality might be improved in elderly people in LMICs. Specifically, they suggest that efforts to ensure universal access to education should confer substantial health benefits and that interventions that target social and economic vulnerability in later life and promote access to effectively organized health care (particularly for stroke) should be considered.

**Additional Information:**

Please access these websites via the online version of this summary at http://dx.doi.org/10.1371/journal.pmed.1001179.

- The World Health Organization provides information on mortality around the world and projections of global mortality up to 2030
- The 10/66 Dementia Research Group is building an evidence base to inform the development and implementation of policies for improving the health and social welfare of older people in LMICs, particularly people with dementia; its website includes background information about demographic and epidemiological aging in LMICs
- Wikipedia has a page on the demographic transition (note: Wikipedia is a free online encyclopedia that anyone can edit; available in several languages)
- Information about the InterVA tool for interpreting verbal autopsy data is available
- The US Centers for Disease Control and Prevention has information about healthy aging

## Introduction

Mortality among older people is a neglected topic in global health. In 2005, 30.2 million of the 58.8 million deaths worldwide occurred in people aged 60 y and older, accounting for 84% of deaths in high income countries, 61% in middle income countries, and 33% in low income countries [1]. Seventy-six percent of the deaths among older people occurred in low and middle income countries (LMICs) [1], where chronic diseases are fast replacing communicable diseases as the leading causes of death and disability [2]. For these countries, the study of old-age mortality trends and their life course determinants is becoming increasingly relevant [3].

In Europe, the protective effects of better education and home ownership upon mortality seem to persist into old age, although with attenuation of the gradient from middle into late life [4]. These findings may not generalise to LMICs, where the social patterning of disease is complex, and evolving rapidly with the epidemiologic and demographic transitions. In settings in the early stages of transition, cardiometabolic risk factors are often found to be more prevalent among the better educated and most affluent [5]. The inversion of social gradients as the less advantaged become more exposed is likely to be an important driver of the epidemic of chronic diseases [6],[7]. On the other hand, inequities in access to health care, which are particularly pronounced for older people in many LMICs [8], favour those with higher status and more resources. Findings from the few population-based studies of determinants of mortality among older people in LMICs are somewhat inconsistent. In Wuhan, China [9], less education, fewer household assets, and financial strain were univariately associated with mortality risk, but only education was significantly associated in the fully adjusted model. In the China Healthy Longevity Longitudinal Survey neither parental occupational status nor the index older person's educational level or occupational attainment was associated with mortality [10]. However, “economic independence” in late life (adequacy of personal economic resources) was strongly inversely associated. In Bambui, Brazil, educational level was inversely associated with mortality risk [11]. In Matlab, Bangladesh, having at least some education and having some household assets were independently associated with lower mortality for both sexes [12]. In the 1998–1999 Indian National Family Health Survey, in contrast to the pattern observed for younger adults, caste had a stronger effect on mortality rates for those aged 65 y and over than did standard of living [13].

In most countries, particularly those with low and middle incomes, death registration is incomplete, and the quality of information on cause of death is highly variable [14]. This is an important limitation to the development and implementation of policy to improve health, and the use of surveys has been advocated to complement official registration procedures [14]. Verbal autopsy (VA) interviews with key informants (family members, carers, or friends) are an important part of this approach, particularly when medical help-seeking is infrequent, and a high proportion of deaths occur at home [15],[16]. Methodologies for VA have been increasingly refined with a focus on harmonisation, and the World Health Organization recommendations now advocate a more standard and structured interview approach, although the recommendations lack specific guidance on timing, respondent characteristics, and interpretation [17]. While assigning cause of death is still complex and problematic, computer-based probabilistic models that generate cause-specific mortality fractions at the population-level provide a faster, cheaper, and more internally consistent alternative to case-by-case physician interpretation [15],[18].

In this paper we aim to provide a comprehensive overview of patterns of mortality among older people as observed through the follow-up period between baseline and incidence waves of the 10/66 Dementia Research Group catchment area studies in Latin America, India, and China. Specifically, we set out to compare mortality rates; to describe the circumstances and antecedents of death; to analyse, from a life course perspective, the effects of socioeconomic conditions (education, mid-life occupational attainment, and late-life household assets and food insecurity) on mortality risk; and, finally, to describe cause-specific mortality fractions and rank the five main causes of death for each site.

## Methods

### Ethics Statement

The study was approved by local ethical committees in each country and by the King's College London Research Ethics Committee. Participants were recruited following informed signed consent. Those who were unable to provide consent themselves were recruited on the basis of a relative's signed agreement. Illiterate persons were read the information sheet and consent form, and invited to express their consent verbally, which was witnessed.

### Sample

The protocol for the 10/66 Dementia Research Group baseline and incidence waves is described in detail in an open-access publication [19]. One-phase population-based surveys were carried out between 2003 and 2005 of all people aged 65 y and over living in seven urban and three rural sites in seven LMICs. Each site comprised one or more geographically defined catchment areas, chosen purposively and door-knocked to identify all eligible participants aged 65 y and over. Urban sites were selected to comprise mixed or mainly lower socioeconomic status households; exclusively high income or professional districts were excluded. Urban sites were located in Cuba (Havana and Matanzas), Dominican Republic (Santo Domingo), Venezuela (Caracas), Peru (Lima), Mexico (Mexico City), China (Xicheng, Beijing), and India (Chennai). Rural sites—selected to be remote from major population centres, with agriculture and related trades as the main local employment—were located in Peru (Canete Province), Mexico (Morelos State), and China (Daxing, Beijing Province). The original target sample size for each country was between 2,000 and 3,000 individuals. The 10/66 Dementia Research Group protocol for the baseline survey included a clinical interview, an informant interview, and a physical examination. In the incidence wave we sought to trace and re-interview all baseline survey participants. We first called on their residence at baseline, revisiting on up to four occasions. Where the participant was no longer resident, we sought information regarding their vital status (if known) and/or current residence, assisted by having recorded at baseline the names and addresses of three non-coresident friends or family members. Where participants had moved away, we sought to re-interview them, even if they had moved out of the original catchment area, by telephone if necessary. Where a participant had died, we recorded date of death, and completed a VA interview with a suitable key informant.

### Measures

Sociodemographic information was collected at baseline on all participants. For the current analysis we use information on age in years, sex, educational level (coded 1 = none, 2 = did not complete primary, 3 = completed primary, 4 = completed secondary, 5 = completed tertiary education; interviewers coded the highest level applicable), and number of household assets (car, television, refrigerator, telephone, mains electricity, mains water, plumbed toilet). Occupational attainment was ascertained from the answer to the question “What is the best (highest level) job you have ever had?” This was coded into four summary categories from nine possible codings: 1 = professional (manager/administrator, professional, associate professional), 2 = clerical or trade (clerical worker or shop keeper), 3 = skilled or semi-skilled manual worker, 4 = unskilled labourer (labourer or agricultural worker). The occupational attainment of the spouse was substituted if that of the index participant was not coded, or coded at a lower level. Food insecurity was assessed through response to the question “Do you ever go hungry because there is not enough food to eat?” Receipt of an occupational or government pension was ascertained by asking participants to report all sources of income and the amounts received.

### Death/Cause of Death

To obtain information on circumstances of death, and likely cause, we used the World Health Organization's “Standard Verbal Autopsy Questionnaire 3: Death of a Person Aged 15 Years and Above” [20] to interview a key informant, usually a family member of the deceased. The fully structured interview covers information regarding the date and place of death, and medical help-seeking for the final illness, and includes 95 questions concerning signs and symptoms noted during the final illness. Responses to these questions were used to compile a data input file (comprising yes/no indicators) for the InterVA model (version 3.2; http://www.interva.net). InterVA's Bayesian algorithm [15],[21] calculates probabilities that a particular death was due to particular causes, given a set of symptoms and circumstances of death. The model, developed by an expert panel, generates up to three likely causes of death from 35 intentionally relatively broad categories, with probabilities attached [15].

### Analyses

We used the 10/66 Dementia Research Group data archive (mortality 3.4) and Stata 11.0. For each site, we describe the number and proportions of baseline participants for whom vital status was determined, the number of deaths, and the number and proportion of those for whom VA interviews were completed. We calculated crude mortality rates per 1,000 person-years at risk. Age- and sex-specific mortality rates were estimated for each country, by sex and age in 5-y bands, by dividing number of deaths by number of person-years contributed in each age band. Direct standardisation (for age, sex, education, occupational attainment, number of assets, and food insecurity) was used to compare mortality rates among sites, with the whole sample as the standard population. We conducted direct standardisation for age, sex, education, assets, occupational class, and food insecurity by weighting each participant with a sampling probability weight equal to the ratio of the frequency of the occurrence of the combination of standardisation variable strata recorded for that participant in the standard population (all sites combined) and the frequency of the occurrence of this combination in the participant's site. We then estimated the mortality rate per 1,000 person-years for each site, applying weights when summing the numerator and denominator. We used indirect standardisation against US Centers for Disease Control and Prevention national mortality data (applying age- and sex-specific US national mortality rates) [22] to age- and sex-specific person-years of follow-up for each 10/66 Dementia Research Group site to compare the burden of late-life mortality in our samples with that currently experienced in the US, reporting standardised mortality ratios (SMRs) with 95% CIs.

We attempted to assess the possible extent and direction of bias arising from non-ascertainment of vital status at follow-up, by assessing univariate associations between baseline characteristics (age, sex, education, WHODAS 2.0 disability score, and dementia status—all likely mortality risk indicators) and vital status not being ascertained. We then used the same predictors of mortality to predict from a multivariate logistic model the probability of death for each individual (including those whose vital status was not ascertained) during the follow-up period. We then compared the distribution of these probabilities in each site between those whose vital status was and was not ascertained at follow-up.

We used Cox's proportional hazards regression to estimate the effects of age (per 5-y band), sex (men compared with women), education (per level), occupational attainment (per level), number of household assets, food insecurity, and pension receipt (yes versus no) on all cause mortality. We ran two sets of models, one adjusting only for age and sex, and the second mutually adjusting for all seven covariates, separately for each site, and then using a fixed effects meta-analysis to combine coefficients, and Higgin's *I* ^2^ to estimate the degree of heterogeneity, with approximate 95% CIs. Where heterogeneity was statistically significant we also present pooled estimates from random effects models. Given previous evidence for attenuation of socioeconomic gradients with increasing age, we tested for interactions with age for all socioeconomic indicators. We also tested for interactions of sex by education and sex by occupational attainment, since these exposures might have different life course implications for men and women. All estimates were adjusted for household clustering and accompanied by robust 95% CIs.

We compared cause-specific mortality fractions for the InterVA causes relevant to older people, by site, describing the estimated numbers and proportions of deaths by cause. For this purpose, we generated a “cause level” output data file in which each deceased person had up to four records, up to three for each cause and a residual “indeterminate” category, with a weight corresponding to their estimated probability. Thus, the sum of the weights equalled the number of cases, and all descriptive analyses on cause of death were weighted appropriately. To compare the five leading causes of death across sites we further aggregated causes, guided by International Classification of Diseases 10 categories [18], combining acute with chronic cardiac death (heart disease), pneumonia/sepsis with acute respiratory disease (respiratory infection), and suicide with homicide, transport-related accident, and other fatal accident (external causes).

## Results

The cohort at baseline comprised 13,924 participants at risk (Tables 1 and S1). The median follow-up period ranged from 2.8 to 5.0 y by site, with a total of 47,438 person-years of observation. The vital status of 12,373 participants (88.9%) was determined, with 2,306 deaths occurring during the follow-up period, for which 2,138 VA interviews were completed. The proportion deceased at follow-up was higher in China, the Dominican Republic, and Cuba than in other countries (in part a function of the longer follow-up interval in those sites). The mean age at baseline was lower in the sites in Venezuela, rural China, and urban India. Women preponderated in all sites. Levels of education were notably lower in the sites in the Dominican Republic, Mexico, India, and rural China, where half to three-quarters had not completed primary education. Occupational attainment was lowest in the Dominican Republic, and in the rural sites in Peru, Mexico, and China. The prevalence of food insecurity was highest in the sites in the Dominican Republic, rural Peru, rural Mexico, and India, and pension coverage was particularly limited in the sites in the Dominican Republic, rural Mexico, rural China, and urban India.

**Table 1:** Cohort characteristics.

Characteristic	Site										
	Cuba	Dominican Republic	Venezuela	Peru (Urban)	Peru (Rural)	Mexico (Urban)	Mexico (Rural)	China (Urban)	China (Rural)	India (Urban)	All Sites Combined
**Baseline sample**	2,813	2,011	1,997	1,381	552	1,003	1,000	1,160	1,002	1,005	13,924
**Vital status determined (percent of baseline sample)**	2,637 (93.7%)	1,706 (84.8%)	1,697 (84.5%)	1,245 (90.2%)	507 (91.8%)	909 (90.6%)	933 (93.3%)	989 (85.2%)	1,002 (100.0%)	748 (74.4%)	12,373 (88.9%)
**Deaths (percent of those with vital status determined)**	609 (23.1%)	467 (27.4%)	200 (11.8%)	98 (7.9%)	54 (10.6%)	99 (10.9%)	110 (11.8%)	224 (22.6%)	291 (29.0%)	154 (20.6%)	2,306 (18.6%)
**Completed VA interviews (percent of deaths)**	598 (98.2%)	467 (100%)	199 (99.5%)	70 (71.4%)	53 (98.1%)	67 (67.7%)	69 (62.7%)	207 (92.4%)	286 (98.3%)	122 (79.2%)	2,138 (92.7%)
**Person-years of follow-up**	10,852.5	7,448.6	7,031.1	3,592.7	1,764.1	2,667.1	2,689.3	4,630.6	4,563.3	2,198.7	47,437.9
**Median years of follow-up (25th and 75th centile)**	4.2 (3.5–5.0)	5.0 (3.6–5.1)	4.2 (4.0–4.8)	2.8 (2.4–3.4)	3.7 (3.6–3.8)	3.0 (2.9–3.2)	3.0 (2.9–3.1)	4.9 (4.6–5.3)	4.9 (4.4–5.2)	2.9 (2.5–3.6)	3.9 (3.0–4.9)
**Mean age at baseline (standard deviation)**	75.2 (7.1)	75.4 (7.6)	72.3 (6.8)	75.0 (7.4)	74.1 (7.3)	74.4 (6.6)	74.1 (6.6)	74.1 (6.3)	72.4 (6.0)	71.4 (6.1)	74.1 (7.0)
**Female sex (percent)**	1,714 (65.0%)	1,130 (66.3%)	1,072 (63.2%)	805 (64.7%)	270 (53.2%)	605 (66.5%)	569 (60.9%)	560 (56.6%)	556 (55.5%)	422 (57.2%)	7,703 (62.3%)
**Did not complete primary education (percent)**	661 (25.1%)	1,211 (71.7%)	499 (30.0%)	114 (9.2%)	206 (41.3%)	530 (58.4%)	787 (84.2%)	346 (35.0%)	693 (69.2%)	492 (66.0%)	5,539 (45.1%)
**Occupational attainment**											
Professional (percent)	952 (38.4%)	50 (2.9%)	541 (34.9%)	552 (45.5%)	46 (9.1%)	189 (20.8%)	27 (2.9%)	518 (52.6%)	40 (4.0%)	102 (14.6%)	3,017 (25.2%)
Trade (percent)	355 (14.3%)	90 (5.3%)	376 (24.3%)	248 (20.4%)	34 (6.7%)	156 (17.1%)	42 (4.5%)	51 (5.2%)	2 (0.2%)	100 (14.3%	1,454 (12.1%)
Skilled labourer (percent)	718 (29.0%)	282 (16.6%)	563 (36.4%)	336 (27.7%)	95 (18.8%)	294 (32.3%)	280 (29.9%)	330 (33.5%)	16 (1.6%)	234 (33.4%)	3,146 (26.3%)
Labourer (percent)	454 (18.3%)	1,276 (75.1%)	68 (4.4%)	78 (6.4%)	331 (65.4%)	271 (29.8%)	586 (62.7%)	85 (8.6%)	944 (94.2%)	265 (37.8%)	4,357 (36.4%)
**Median assets (25th and 75th centile)**	6 (5–6)	5 (4–6)	6 (6–7)	6 (6–6)	5 (4–6)	6 (6–7)	4 (3–6)	5 (5–6)	6 (5–7)	4 (3–5)	6 (5–6)
**Food insecurity (percent)**	130 (5.0%)	208 (12.3%)	87 (5.4%)	56 (4.6%)	67 (13.3%)	36 (4.0%)	82 (8.8%)	0 (0.0%)	12 (1.2%)	147 (19.7%)	825 (6.8%)
**In receipt of a pension (percent)**	2,169 (82.3%)	521 (30.5%)	994 (58.6%)	830 (66.7%)	334 (65.9%)	670 (73.7%)	233 (25.0%)	893 (90.3%)	38 (3.8%)	95 (12.7%)	6,777 (54.8%)

### Circumstances of Death

In the sites in rural Peru and urban China, most deaths (86.8% and 70.1%, respectively) occurred in hospital, whereas in rural China, India, and rural Mexico, the large majority of deaths occurred at home (91.3%, 86.3%, and 65.2%, respectively); in other sites deaths were more evenly distributed between home and health care settings (Table 2). Other than in the India site, the large majority of individuals had received some treatment for their terminal illness (79.5% to 97.5%, by site). Other than for sites in rural Peru and India, home was nearly as frequently, if not more frequently, mentioned as hospital, as a venue for medical care. Community clinics were comparatively rarely used.

**Table 2:** Circumstances of death, by site.

Circumstance	Site										
	Cuba	Dominican Republic	Venezuela	Peru (Urban)	Peru (Rural)	Mexico (Urban)	Mexico (Rural)	China (Urban)	China (Rural)	India	Total
**Completed VA interviews**	598	467	199	70	53	67	69	207	286	122	2,138
**Place of death**											
Home, *n* (percent)	282 (47.6%)	262 (57.8%)	78 (40.8%)	37 (52.9%)	7 (13.2%)	38 (56.7%)	45 (65.2%)	62 (29.9%)	261 (91.3%)	88 (86.3%)	1,160 (55.5%)
Hospital or other health facility, *n* (percent)	299 (50.5%)	182 (40.2%)	108 (56.6%)	32 (45.7%)	46 (86.8%)	29 (43.3%)	23 (33.3%)	145 (70.1%)	23 (8.0%)	14 (13.7%)	901 (43.1%)
Other, *n* (percent)	11 (1.9%)	9 (2.0%)	5 (2.6%)	1 (1.4%)	0	0	1 (1.4%)	0	2 (0.7%)	0	29 (1.4%)
MV	6	14	8	0	0	0	0	0	0	20	48
**Treatment for illness leading to death**											
Received treatment, *n* (percent)	473 (79.5%)	363 (80.1%)	163 (83.2%)	63 (90.0%)	27 (90.0%)	61 (91.0%)	62 (89.9%)	196 (95.6%)	272 (97.5%)	24 (22.4%)	1,704 (79.7%)
MV	3	14	3	0	23	0	0	2	7	15	67
Underwent surgery, *n* (percent)	55 (9.3%)	39 (8.6%)	26 (13.3%)	3 (4.3%)	1 (1.9%)	13 (19.4%)	11 (15.9%)	3 (1.5%)	4 (1.4%)	8 (7.9%)	163 (7.8%)
MV	5	16	4	0	23	0	0	2	8	21	56
**Places of treatment (not mutually exclusive)**											
Home, *n* (percent)	275 (46.9%)	202 (46.4%)	122 (64.6%)	43 (62.3%)	3 (5.7%)	24 (35.8%)	37 (53.6%)	119 (58.3%)	180 (64.5%)	1 (0.8%)	1,006 (49.5%)
MV	12	32	10	1	23	0	0	3	7	17	105
Clinic, *n* (percent)	83 (14.2%)	93 (21.1%)	87 (46.3%)	10 (14.5%)	0	23 (34.3%)	29 (42.0%)	55 (27.1%)	35 (12.5%)	7 (6.7%)	422 (20.7%)
MV	12	26	11	1	23	0	0	4	7	17	101
Hospital, *n* (percent)	339 (57.6%)	238 (54.2%)	77 (41.6%)	41 (58.6%)	23 (76.7%)	36 (53.7%)	49 (71.0%)	143 (70.1%)	155 (55.6%)	13 (12.4%)	1,114 (49.5%)
MV	9	28	14	14	23	0	0	3	7	17	105

MV, missing values.

### Mortality Rates

**Figure 1:**
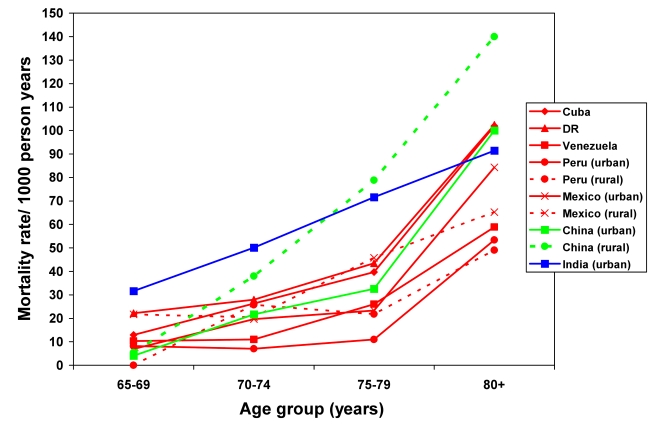
Mortality rate (per 1,000 person-years) by age group for each site among women. DR, Dominican Republic.

**Figure 2:**
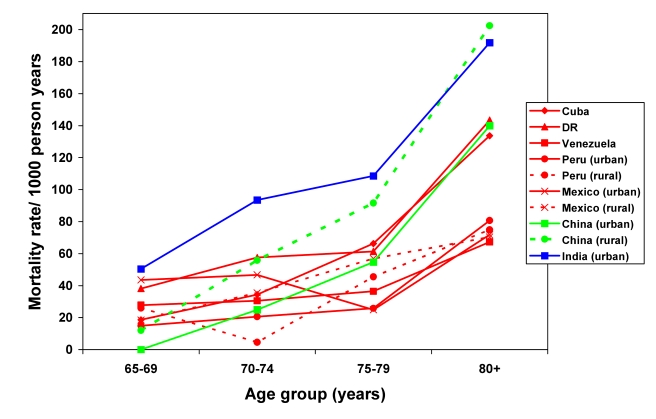
Mortality rate (per 1,000 person-years) by age group for each site among men. DR, Dominican Republic.

Crude mortality rates varied from 27.3 (urban Peru) to 70.0 (urban India) per 1,000 person-years. Rates were higher among men in all centres and tended to increase exponentially with increasing age for both sexes (Figures 1 and 2; Table S2). In countries where both rural and urban sites were sampled, rates tended to be slightly higher in rural settings. Direct standardisation for age, sex, education, occupational attainment, and number of assets had little effect on the variation among sites, with standardised rates ranging from 20 (rural Peru) to 60 (rural China and urban India) per 1,000 person-years (Table 3). After indirect standardisation, applying age- and sex-specific US national population mortality rates [22], mortality was higher than in the US in the 10/66 Dementia Research Group sites in urban India (SMR 198, 95% CI 168–332), rural China (SMR 162, 95% CI 145–182), the Dominican Republic (SMR 124, 95% CI 113–136), and Cuba (SMR 112, 95% CI 104–112); similar in urban China; but significantly lower in urban Peru (SMR 57, 95% CI 46–69), rural Peru (SMR 63, 95% CI 48–82), Venezuela (SMR 71, 95% CI 62–82), and urban Mexico (SMR 82, 95% CI 67–99).

**Table 3:** Total and sex-specific mortality rates (per 1,000 person-years) among people aged 65 and over and standardized mortality ratio.

Site	Total Mortality			Sex-Specific Mortality		
	Total Crude Mortality Rate	Standardized Mortality Rate^a^	SMR^b^	Sex	Sex-Specific Crude Mortality Rate	SMR^b^
**Cuba** (*n* = 2,637)	56.1 (51.8–60.7)	50.5 (46.5–54.8)	112 (104–122)	Female	51.2 (46.1–56.6)	109 (99–121)
				Male	65.9 (58.0–74.5)	118 (104–134)
**Dominican Republic** (*n* = 1,706)	62.7 (57.2–68.6)	43.8 (39.4–48.6)	124 (113–136)	Female	55.0 (48.8–61.8)	116 (103–130)
				Male	78.9 (68.4–90.8)	139 (120–160)
**Peru (urban)** (*n* = 1,245)	27.3 (22.3–33.1)	20.2 (17.3–23.7)	57 (46–69)	Female	20.2 (15.0–26.6)	48 (36–64)
				Male	40.4 (30.4–52.7)	67 (50–87)
**Peru (rural)** (*n* = 507)	30.6 (23.2–39.6)	21.4 (17.2–26.2)	63 (48–82)	Female	23.3 (14.9–34.6)	56 (36–85)
				Male	39.1 (27.2–54.5)	68 (47–95)
**Venezuela** (*n* = 1,706)	28.4 (24.7–32.6)	27.1 (20.6–34.8)	71 (62–82)	Female	23.1 (19.0–27.9)	64 (52–77)
				Male	36.9 (30.0–44.9)	81 (66–99)
**Mexico (urban)** (*n* = 910)	37.1 (30.3–45.0)	33.3 (27.1–40.2)	82 (67–99)	Female	32.3 (24.7–41.4)	82 (63–105)
				Male	47.1 (34.2–63.3)	82 (60–110)
**Mexico (rural)** (*n* = 935)	40.9 (33.8–49.1)	31.6 (25.6–38.2)	87 (72–105)	Female	36.9 (28.5–47.1)	93 (72–119)
				Male	47.3 (35.4–62.0)	81 (61–106)
**China (urban)** (*n* = 980)	48.4 (42.3–55.0)	38.8 (33.6–44.5)	102 (89–116)	Female	39.8 (32.7–47.9)	97 (80–117)
				Male	60.0 (49.9–71.6)	106 (88–127)
**China (rural)** (*n* = 1,002)	63.8 (56.8–71.4)	59.7 (53.1–67.0)	162 (145–182)	Female	59.4 (50.5–69.5)	163 (139–191)
				Male	69.2 (58.4–81.4)	162 (136–190)
**India** (*n* = 748)	70.0 (59.6–81.8)	59.9 (50.6–71.0)	198 (168–232)	Female	50.5 (39.2–64.1)	173 (135–220)
				Male	92.8 (74.4–114.5)	223 (178–274)

Rate and ratio data are given as percent (95% CI).

a Standardized by age group, sex, education, occupational attainment, household assets, and food insecurity, using the whole sample as the standard population.

b Standardized for age group and sex to US national rates for 2005.

### Potential Bias Arising from Non-Ascertainment of Vital Status

Of the likely predictors of mortality (baseline age, sex, education, WHODAS 2.0 disability score, and dementia), sex and disability were not associated with ascertainment of vital status at follow-up in any site (Table S3). Those with more education at baseline and younger participants were more likely not to be traced in the urban China site. Those with dementia at baseline were more likely not to have their vital status ascertained in the urban Mexico site (Table S3). The predicted probability of death by follow-up, derived from logistic regression using all of the above predictors, was similar for all sites between those who were and were not followed-up, other than in the urban Mexican site (24% higher predicted probability of death among those in whom vital status was not ascertained) and urban China (24% lower predicted probability of death among those in whom vital status was not ascertained) (Table S4).

### Demographic and Socioeconomic Predictors of Mortality

Older age was significantly associated with mortality in all sites, whether adjusted for sex alone (Table 4), or for sex and socioeconomic indicators (Table 5). There was substantial and significant heterogeneity (*I* ^2^ = 72%) in the size of this effect across sites; in the fully adjusted model the random effects pooled mortality hazard increased by 1.57 (95% CI 1.47–1.67) for each 5-y age band. The effect of male sex was statistically significant in all sites other than rural Peru and rural Mexico, with minimal heterogeneity, and was, if anything, slightly enhanced after controlling for socioeconomic indicators (fully adjusted pooled hazard ratio [HR] 1.59, 95% CI 1.45–1.74). Adjusting for age and sex, it was found that less education, lower occupational attainment, and food insecurity were each associated with increased mortality risk after pooling coefficients across sites, with no or minimal heterogeneity of effect among sites (Table 4). Heterogeneity was more pronounced for household assets (*I* ^2^ = 47%), with protective effects of more assets being mostly apparent in the sites in Cuba and the Dominican Republic, and for pension receipt (*I* ^2^ = 39%), where despite a generally protective effect, the 3.8% of individuals receiving a pension in rural China had an increased mortality (HR 1.97, 95% CI 1.16–3.36). After mutual adjustment for each of the other socioeconomic indicators in the fully adjusted model, the effects were all slightly attenuated (Table 5). After pooling, there was still evidence of an independent, consistent protective effect of more education (pooled HR 0.93, 95% CI 0.89–0.98). There was no evidence for effect modification by age for any of the socioeconomic indicators: education (pooled HR 1.03, 95% CI 0.99–1.07, *I* ^2^ = 8%), occupational attainment (1.02, 95% CI 0.98–1.07, *I* ^2^ = 0%), assets (1.01, 95% CI 0.98–1.04, *I* ^2^ = 63%), or food insecurity (0.87, 95% CI 0.76–1.00, *I* ^2^ = 0%). Neither was there evidence that the effects of education (pooled HR for interaction term 1.04, 95% CI 0.96–1.13, *I* ^2^ = 33%) or occupational attainment (0.95, 95% CI 0.87–1.05, *I* ^2^ = 0%) were modified by sex.

**Table 4:** Age- and sex-adjusted hazard ratios for the associations between sociodemographic factors and mortality, by site.

Site/Statistical Test	Sociodemographic Factor						
	Age (by 5-y Band)	Sex (Male versus Female)	Education (per Level)	Occupational Attainment (per Level)	Assets (per Asset)	Food Insecurity	In Receipt of a Pension
**Site**							
Cuba	1.61 (1.53–1.70)	1.46 (1.24–1.72)	0.91 (0.84–0.99)	1.09 (1.02–1.18)	0.88 (0.81–0.95)	1.23 (0.88–1.71)	0.96 (0.77–1.21)
Dominican Republic	1.47 (1.38–1.56)	1.56 (1.29–1.87)	0.94 (0.85–1.03)	1.26 (1.09–1.46)	0.84 (0.79–0.89)	1.15 (0.87–1.52)	0.89 (0.73–1.09)
Peru (urban)	1.79 (1.64–2.06)	1.76 (1.20–2.58)	0.84 (0.69–1.03)	1.15 (0.94–1.40)	1.12 (0.80–1.55)	1.32 (0.63–2.78)	1.09 (0.69–1.71)
Peru (rural)	1.64 (1.55–1.94)	1.37 (0.78–2.41)	0.81 (0.62–1.05)	1.43 (0.99–2.08)	1.00 (0.83–1.20)	1.63 (0.84–3.18)	0.89 (0.50–1.58)
Venezuela	1.53 (1.39–1.67)	1.72 (1.31–2.27)	0.81 (0.68–0.97)	1.03 (0.88–1.22)	0.99 (0.87–1.12)	1.14 (0.62–2.09)	0.74 (0.55–0.99)
Mexico (urban)	1.55 (1.35–1.79)	1.39 (0.93–2.07)	0.92 (0.76–1.12)	1.16 (0.97–1.38)	0.98 (0.84–1.14)	0.82 (0.28–2.43)	0.78 (0.47–1.32)
Mexico (rural)	1.48 (1.30–1.68)	1.18 (0.82–1.72)	0.85 (0.67–1.07)	1.09 (0.83–1.43)	0.93 (0.83–1.03)	1.36 (0.74–2.49)	0.77 (0.50–1.19)
China (urban)	1.98 (1.79–2.20)	1.35 (1.04–1.75)	0.92 (0.82–1.02)	1.02 (0.90–1.15)	0.92 (0.75–1.12)	None exposed	0.63 (0.41–0.96)
China (rural)	1.69 (1.56–1.84)	1.43 (1.13–1.80)	0.91 (0.79–1.05)	0.95 (0.79–1.14)	0.95 (0.88–1.04)	1.47 (0.56–3.88)	1.97 (1.16–3.36)
India	1.39 (1.24–1.55)	1.76 (1.27–2.43)	0.91 (0.76–1.04)	1.06 (0.90–1.26)	1.01 (0.91–1.12)	1.14 (0.77–1.70)	0.82 (0.50–1.35)
**Chi square test for heterogeneity**	36.8, 9 df, *p*<0.001	5.4, 9 df, *p* = 0.79	3.6, 9 df, *p* = 0.93	10.1, 9 df, *p* = 0.34	17.1, 9 df, *p* = 0.05	1.9, 8 df, *p* = 0.98	14.7, 9 df, *p* = 0.10
**Higgins ** ***I*** **^2^ (95% CI)**	76 (55–87)	0 (0–62)	0 (0–62)	0 (0–52)	47 (0–75)	0 (0–65)	39 (0–71)
**Pooled fixed effect**	1.59 (1.54–1.63)	1.49 (1.38–1.62)	0.90 (0.87–0.94)	1.09 (1.04–1.14)	0.91 (0.88–0.94)	1.21 (1.03–1.42)	0.89 (0.80–0.99)
**Pooled random effect**	1.60 (1.51–1.70)				0.93 (0.89–0.98)		

Site-specific values are HR (95% CI).

df, degrees of freedom.

**Table 5:** Mutually adjusted hazard ratios for the associations between sociodemographic factors and mortality, by site.

Site/Statistical Test	Sociodemographic Factor						
	Age (by 5-y Band)	Sex (Male versus Female)	Education (per Level)	Occupational Attainment (per Level)	Assets (per Asset)	Food Insecurity	In Receipt of a Pension
**Site**							
Cuba	1.60 (1.51–1.70)	1.49 (1.25–1.78)	0.93 (0.85–1.03)	1.02 (0.94–1.11)	0.92 (0.85–1.00)	1.13 (0.79–1.62)	0.93 (0.72–1.19)
Dominican Republic	1.48 (1.38–1.57)	1.61 (1.32–1.96)	1.04 (0.93–1.15)	1.21 (1.04–1.42)	0.85 (0.80–0.90)	0.99 (0.75–1.32)	0.96 (0.78–1.18)
Peru (urban)	1.72 (1.46–2.02)	1.96 (1.29–2.98)	0.89 (0.71–1.11)	1.08 (0.87–1.33)	1.11 (0.81–1.52)	1.48 (0.70–3.16)	1.17 (0.71– 1.92)
Peru (rural)	1.60 (1.35–1.89)	1.63 (0.88–3.03)	0.78 (0.58–1.06)	1.34 (0.90–1.98)	1.12 (0.91–1.39)	1.75 (0.87–3.51)	0.76 (0.41–1.42)
Venezuela	1.38 (1.23–1.55)	1.99 (1.45–2.73)	0.81 (0.66–0.99)	0.94 (0.79–1.12)	0.97 (0.83–1.12)	1.21 (0.65–2.24)	0.88 (0.64–1.23)
Mexico (urban)	1.58 (1.36–1.84)	1.41 (0.94–2.13)	0.99 (0.78–1.26)	1.15 (0.93–1.42)	0.99 (0.84–1.16)	0.76 (0.25–2.36)	0.77 (0.45–1.30)
Mexico (rural)	1.45 (1.27–1.66)	1.18 (0.81–1.72)	0.89 (0.68–1.15)	1.05 (0.79–1.40)	0.93 (0.82–1.05)	1.28 (0.69–2.38)	0.72 (0.46–1.14)
China (urban)	1.94 (1.74– 2.17)	1.59 (1.16–2.17)	0.94 (0.82–1.09)	0.99 (0.85–1.16)	0.96 (0.78–1.19)	None exposed	0.70 (0.44–1.12)
China (rural)	1.67 (1.53–1.82)	1.45 (1.13–1.87)	0.88 (0.76–1.02)	1.16 (0.88–1.52)	0.96 (0.88–1.04)	1.31 (0.49–3.49)	3.08 (1.57–6.05)
India	1.37 (1.22–1.55)	2.15 (1.50–3.10)	0.87 (0.71–1.07)	1.01 (0.83–1.24)	1.08 (0.93–1.24)	1.23 (0.80–1.90)	0.91 (0.53–1.58)
**Chi square test for heterogeneity**	32.6, 9 df, *p*<0.001	9.3, 9 df, *p* = 0.41	8.8, 9 df, *p* = 0.46	8.4, 9 df, *p* = 0.50	17.5, 9 df, *p* = 0.04	3.7, 8 df, *p* = 0.88	16.7, 9 df, *p* = 0.05
**Higgins ** ***I*** **^2^ (95% CI)**	72 (48–85)	3 (0–64)	0 (0–62)	0 (0–62)	49 (0–75)	0 (0–65)	46 (0–74)
**Pooled fixed effect**	1.57 (1.52–1.62)	1.59 (1.45–1.74)	0.93 (0.89–0.98)	1.05 (1.00–1.11)	0.93 (0.89–0.96)	1.15 (0.97–1.36)	0.93 (0.83–1.05)
**Pooled random effect**	1.57 (1.47–1.67)				0.95 (0.90–1.01)		0.93 (0.78–1.11)

Site-specific values are HR (95% CI).

df, degrees of freedom.

### Cause of Death

Table 6 describes the estimated cause-specific mortality fractions for each site for the 21 causes generated by the InterVA algorithm. The proportion of indeterminate causes ranged from 21.7% to 28.1% in the Latin American sites, but was somewhat higher in urban China (31.7%), rural China (32.6%), and India (41.8%). The five leading causes of death for each site are listed in Table 7. Stroke was by far the most common cause overall (21.4%), and was the leading or joint-leading cause of death in all sites other than rural Peru (third leading cause) and rural Mexico (fourth leading cause). Heart disease (7.4% of all deaths) was also among the leading causes of death in all sites other than Mexico, rural China, and India. Diabetes (6.1% of all deaths) was among the leading causes in all sites other than the Dominican Republic, rural Peru, and rural China. Chronic diseases as a whole (stroke, heart disease, diabetes, chronic respiratory disease, and malignancy) accounted for the majority of deaths where the cause was determined in all sites other than rural Peru (23.6% of all deaths and 31.7% of determined deaths). Other important causes of death included liver disease (accounting for 10% or more of deaths in rural Peru, Venezuela, and urban and rural Mexico) and tuberculosis (accounting for 10% or more of deaths in rural Peru, urban Mexico, and urban and rural China and India).

**Table 6:** Cause-specific mortality fractions as interpreted probabilistically by the InterVA model for each site.

Cause of Death	Site										
	Cuba	Dominican Republic	Venezuela	Peru (Urban)	Peru (Rural)	Mexico (Urban)	Mexico (Rural)	China (Urban)	China (Rural)	India	Total
Indeterminate	135 (22.6)	131 (28.1)	53 (26.8)	18 (25.7)	13 (25.5)	16(23.9)	15 (21.7)	65 (31.7)	93 (32.6)	51 (41.8)	590 (27.7)
Stroke	113 (18.9)	109 (23.4)	32 (15.2)	20 (28.6)	7 (13.7)	10 (14.9	5 (7.2)	59 (28.8)	83 (29.1)	20 (16.4)	456 (21.4)
Acute cardiac death	51 (8.5)	14 (3.0)	8 (4.0)	2 (2.9)	1 (2.0)	2 (3.0)	1 (1.4)	5 (2.4)	3 (1.1)	3 (2.5)	90 (4.2)
Chronic cardiac death	31 (5.2)	12 (2.6)	15 (7.6)	1 (1.4)	1 (2.0)	1 (1.5)	1 (1.4)	5 (2.4)	1 (0.4)	1 (0.8)	69 (3.2)
Diabetes	37 (6.2)	21 (4.5)	11 (5.5)	4 (5.7)	2 (3.9)	5 (7.5)	6 (8.7)	22 (10.7)	9 (3.2)	12 (9.8)	129 (6.1)
Acute respiratory disease	25 (4.2)	2 (0.4)	1 (0.5)	2 (2.9)	0	0	0	0	1 (0.4)	0	31 (1.5)
Chronic respiratory disease	22 (3.7)	7 (1.5)	9 (4.5)	2 (2.9)	1 (2.0)	6 (9.0)	4 (5.8)	12 (5.9)	23 (8.1)	1 (0.8)	87 (4.1)
Pneumonia/sepsis	61 (10.2)	24 (5.2)	6 (3.0)	7 (10.0)	4 (7.8)	1 (1.5)	2 (2.9)	2 (1.0)	9 (3.2)	9 (7.1)	125 (5.9)
Tuberculosis	22 (3.7)	38 (8.2)	14 (7.1)	3 (4.3)	8 (15.7)	8(11.9)	6 (8.7)	24 (11.7)	40 (14.0)	13 (10.7)	176 (8.3)
Malignancy	19 (3.2)	21 (4.5)	4 (2.0)	1 (1.4)	0	1 (1.5)	1 (1.4)	1 (0.5)	1 (0.4)	1 (0.8)	50 (2.3)
Suicide	1 (0.2)	0	0	0	0	0	0	0	0	0	1 (0.0)
Homicide	1 (0.2)	0	0	0	0	0	0	0	0	0	1 (0.0)
Transport-related accident	2 (0.3)	1 (0.2)	1 (0.5)	0	0	0	1 (1.4)	2 (1.0)	2 (0.7)	5 (4.1)	14 (0.7)
Other fatal accident	6 (1.0)	15 (3.2)	2 (1.0)	3 (4.3)	2 (3.9)	3 (4.5)	2 (2.9)	0	1 (0.4)	4 (3.3)	38 (1.8)
Kidney/urinary disease	6 (1.0)	6 (1.3)	2 (1.0)	1 (1.4)	2 (3.9)	1 (1.5)	1 (1.4)	1 (0.5)	2 (0.7)	0	22 (1.0)
Liver disease	39 (6.5)	46 (9.9)	27 (13.6)	3 (4.3)	8 (15.7)	10 (14.9)	20 (29.0)	3 (1.5)	11 (3.9)	1 (0.8)	168 (7.9)
Bloody diarrhoea	3 (0.5)	0	0	0	1 (2.0)	0	0	0	0	0	4 (0.2)
Malnutrition	2 (0.3)	4 (0.9)	2 (1.0)	0	0	0	0	0	1 (0.4)	0	9 (0.4)
Other digestive disease	5 (0.8)	2 (0.4)	1 (0.5)	0	0	0	1 (1.4)	1 (0.5)	1 (0.4)	0	11 (0.5)
HIV/AIDS-related death	0	6 (1.3)	7 (3.5)	0	0	1 (1.5)	2 (2.9)	0	0	0	16 (0.8)
Meningitis	2 (0.3)	1 (0.2)	2 (1.0)	0	0	0	0	1 (0.5)	2 (0.7)	0	8 (0.4)
Tetanus	15 (2.5)	6 (1.3)	3 (1.5)	3 (4.3)	1 (2.0)	2 (3.0)	1 (1.4)	2 (1.0)	2 (0.7)	1 (0.8)	36 (1.7)
Weighted total	598 (100)	466 (100)	198 (100)	70 (100)	51 (100)	67 (100)	69 (100)	205 (100)	285 (100)	122 (100)	2,131 (100)
**Proportion arising from chronic disease causes** a											
Percentage of all deaths	45.7%	39.5%	38.8%	42.9%	23.6%	37.4%	47.6%	50.7%	42.3%	31.1%	41.3%
Percentage of deaths where cause was determined	59.0%	54.9%	53.0%	57.7%	31.7%	49.1%	60.8%	74.2%	62.8%	53.4%	57.1%

Cause-specific mortality data are given as *n* (percent).

a Chronic diseases were taken to comprise stroke, acute cardiac death, chronic cardiac death, diabetes, chronic respiratory disease, and malignancy.

**Table 7:** Five leading causes of death by site, using cause of death as interpreted probabilistically by the InterVA model.

Site	Ranked Cause of Death				
	1	2	3	4	5
**Cuba**	Stroke (18.9%)	Acute respiratory infection (14.4%)	Heart disease (13.7%)	Liver disease (6.5%)	Diabetes (6.2%)
**Dominican Republic**	Stroke (23.4%)	Liver disease (9.9%)	Tuberculosis (8.2%)	Heart disease (5.6%), acute respiratory infection (5.6%)	
**Peru (urban)**	Stroke (28.6%)	Acute respiratory infection (12.9%)	Diabetes (5.7%)	Heart disease (4.3%), tuberculosis (4.3%), liver disease (4.3%), external causes (4.3%), tetanus (4.3%)	
**Peru (rural)**	Tuberculosis (15.7%), liver disease (15.7%)		Stroke (13.7%)	Acute respiratory infection (7.8%)	Heart disease (4.0%)
**Venezuela**	Stroke (15.2%)	Liver disease (13.6%)	Heart disease (11.6%)	Tuberculosis (7.1%)	Diabetes (5.5%)
**Mexico (urban)**	Stroke (14.9%), liver disease (14.9%)		Tuberculosis (11.9%)	Chronic respiratory disease (9.0%)	Diabetes (7.5%)
**Mexico (rural)**	Liver disease (29.0%)	Diabetes (8.7%), tuberculosis (8.7%)		Stroke (7.2%)	Chronic respiratory infection (5.8%)
**China (urban)**	Stroke (28.8%)	Tuberculosis (11.7%)	Diabetes (10.7%)	Chronic respiratory disease (5.9%)	Heart disease (4.8%)
**China (rural)**	Stroke (29.1%)	Tuberculosis (14.0%)	Chronic respiratory disease (8.1%)	Liver disease (3.9%)	Acute respiratory infection (3.6%)
**India**	Stroke (16.4%)	Tuberculosis (10.7%)	Diabetes (9.8%)	External causes (7.4%)	Acute respiratory infection (7.1%)

## Discussion

In this catchment area population-based longitudinal study of mortality among older people, there was a 2.6-fold variation in crude rates between sites. Lower levels of education and occupational attainment in earlier life, fewer household assets, food insecurity, and not receiving a pension in later life were associated with mortality; less education remained independently associated after mutual adjustment. There was still a 3-fold variation in mortality rates among sites after standardising for compositional differences in age, sex, and all socioeconomic indicators. Compared with national age- and sex-specific rates in the US, rates in India and rural China were substantially higher, and rates in Peru and Venezuela substantially lower. In most sites a substantial minority or small majority of older people died at home. Nevertheless, other than in India, most had sought some medical attention in the course of their final illness, which tended to be provided at home or in hospital, rather than in a community health centre. Chronic diseases, most particularly stroke, heart disease, and diabetes, were the leading causes of death in all sites other than rural Peru.

### Strengths and Limitations

The main strengths of this study are the successful application of a uniform methodology to ascertain deaths and determine cause of death across urban and rural catchment area sites in Latin America, China, and India. The baseline survey data allowed us to study, prospectively, the effects of a range of potentially relevant social determinants. Although we did not keep the catchment area populations under continuous surveillance, we were able to determine the vital status of 12,373 of 13,924 (88.9%) baseline participants, identifying 2,306 deaths among them, of which 2,138 (92.7%) had completed VA interviews. A significant limitation is that our samples are not nationally representative, and hence our findings refer to the catchment areas studied and may not generalise to other urban or rural locations in the countries concerned. Although the study was prospective, education and occupational attainment exposures were recalled and may have been subject to random error and information bias. Older people who had become frail or unwell may have moved to live with children or other relatives; the household assets that were recorded may not therefore have reflected those that they were generally exposed to in later life [3],[23]. Likewise, food insecurity may have arisen as a consequence rather than as a cause of ill health leading to death. Our selection of predominately lower socioeconomic status or mixed neighbourhoods for our catchment areas may have somewhat constrained the variance of socioeconomic exposures, leading to underestimation of their effects.

The low mortality rates observed in sites in Peru, Venezuela, and urban Mexico may be due to underascertainment, to the extent that deaths were overrepresented among those not traced (and hence for whom vital status could not be determined). The proportion not traced (10%–15%) was not particularly high in these sites. Our supplementary analyses, conducted to explore the likely extent and direction of bias, supported the possibility of an underestimation of mortality rates only in urban Mexico, where dementia at baseline was overrepresented in those not traced, for whom the predicted probability of death was 24% higher than for those who were traced. It is also possible that the low mortality rates may have reflected the particular characteristics of the catchment areas that were selected. These were, by design, predominately lower socioeconomic status communities; however, levels of occupational attainment were somewhat higher in Venezuela and urban Peru than in other sites. In sites in urban China and urban India, the relatively high loss to follow-up was mainly explained by external factors: closure of an apartment block and an urban infrastructure project, respectively. In urban China, indicators of mortality risk, particularly older age and low education, were underrepresented in those who were not traced, suggesting, if anything, a possible overestimation of mortality rates in that site.

### Comparison with Other Research

There are few directly comparable data. In the Bambui study, conducted in a Brazilian rural town with a high prevalence of Chagas disease, the mortality rate for those aged 60 y and over was 48.3 per 1,000 person-years [11], much higher than the rates we observed in sites in Peru, Venezuela, and Mexico, and probably more similar to the rates we observed in Cuba and the Dominican Republic. It is tempting to speculate, but difficult to assess, whether culturally determined lifestyles or Native American ancestry may have played a role. Overall, the lower mortality in Latin America is probably a reflection of the more advanced stages of the health transition process in these areas compared to rural China and India.

Life course approaches to understanding the effect of socioeconomic factors on health have, explicitly or implicitly, tested four main models of social causation [24]: (1) latent effect models (an enduring effect of early-life socioeconomic status independent of later socioeconomic status), (2) pathway models (a “chain of disadvantage”, wherein early-life circumstances influence adult experiences and behaviours), (3) social mobility models (positing an independent effect of changes in social status), and (4) cumulative effect models (wherein the effects of adverse social environments accumulate across the life course). Each is supported to some extent by empirical data, with the strongest evidence for latent and cumulative effects, and the weakest for social mobility [24],[25]. To our knowledge, the only test of the applicability of such models for mortality in older people in LMICs was an analysis of data from the China Healthy Longevity Longitudinal Survey [10]. While the authors inferred support for the latent and cumulative effect models, the core indicators of socioeconomic position, education, and occupational attainment were not associated with mortality. There were latent (independent) protective effects of arm length (as an indicator of early nutritional environment) and having both parents living at the age of 10 y. The striking findings were the dose–response effect of social mobility, and a strong inverse association with the adequacy of socioeconomic resources in late life. In our analyses, consistent with some other studies from LMICs [9],[12], we did find a latent protective effect of education, controlling for occupational attainment and late-life resources (household assets). This was a fairly consistent effect across the wide range of sites studied, with minimal statistical heterogeneity, and amounted to a 7% reduction in mortality hazard per level of education. The pooled effects of mid- and late-life socioeconomic indicators, occupational attainment and assets, and late-life indicators of income security, food insecurity and receiving a pension—all apparent in the age- and sex-adjusted models—were no longer statistically significant in the fully mutually adjusted model. Our findings are therefore broadly consistent with latent effect and pathway models of disadvantage. We lacked appropriate exposure data to explore the effect of social mobility.

The independent associations with food insecurity and receiving a pension in late life, at least in the age- and sex-adjusted models, are consistent with findings from other studies of an association between indicators of economic strain in late life and mortality [9],[10], and suggest a further mechanism—that deterioration in economic circumstances in late life may have a proximal effect on mortality risk. In a more detailed analysis of the determinants of mortality in our India site, we found that undernutrition was an important independent predictor, with a population attributable risk fraction of 0.44 [26]. Lack of social protection for older people, arising from income insecurity and diminishing availability of family support, is a widespread but under-prioritised problem in health and human development agendas [27],[28].

### Cause of Death

The lack of data and poor reliability of existing information on cause-specific mortality from many LMICs is likely to be exacerbated among older people, because of the increased likelihood of complex comorbidity, and hence multiple contributing causes, and the increased likelihood of death occurring at home, sometimes with minimal or no medical attention. However, other than in India, a high proportion of older people who died in our study received some kind of treatment for the disease leading to death. This was often provided at home, and with the notable exceptions of rural Peru and urban China, a high proportion of deaths also occurred at home. We used the VA approach to circumvent anticipated problems with availability and quality of cause of death data from official registration. VAs were completed for 92.7% of all deaths, using the standard structured assessment currently recommended by the World Health Organization, in an attempt to improve international harmonisation [17]. Rather than using physician determination, we submitted indicator data to the InterVA probabilistic algorithm to generate up to three causes of death for each participant. While the validity of this approach is modest for individual determinations (if, rather questionably, physician determination is taken as the “gold standard”) [29], it does seem to return robust, consistent, and reproducible estimates of cause-specific mortality fractions at the population level [15],[29]. As such, it is an appropriate tool for making comparisons between populations and over time. However, we acknowledge that symptom recognition and recall is likely to differ across sites, which might be responsible for some residual variability on main causes of death between sites.

Our main finding was that chronic diseases, particularly stroke, heart disease, and diabetes, were the dominant causes of death in almost all sites. Stroke was by far the most common, ranking first in all sites other than rural Peru and rural Mexico. It is estimated that stroke caused around 5.7 million deaths in 2005 [30], of which more than 87% occurred in LMICs, 83% in individuals who were aged 60 y and over [1]. Stroke is also an important contributor to disability and dependence among older survivors in LMICs [31]–[33]. Chronic liver disease was identified as an important cause of death in rural Peru, Venezuela, and urban and rural Mexico. While there may have been some misattribution, with a failure to detect malignancy and chronic cardiac failure, the regional patterning may be significant. In the US, cirrhosis, chronic liver disease, and liver cancer are recognised to be particularly common causes of death among Hispanics, and in the National Health and Nutrition Examination Survey, Hispanic men and women were both more likely to have elevated aminotransferase activity, an indicator of the burden of liver disease [34]. The reasons for this susceptibility are not fully understood; the seroprevalences of hepatitis B and C virus infection are relatively low by global standards [35].

In common with the Agincourt study in South Africa [15], we found that the InterVA algorithm returned a relatively high proportion of deaths from tuberculosis, particularly in rural Peru, urban Mexico, and urban and rural China and India. Again, the regional patterning is consistent with expectations. A recent register-based study from Hunan Province, China, showed that the prevalence of tuberculosis was over twice as high in those aged 65 y and older than in those aged 15–64 y [36]. Older people accounted for around a fifth of treated cases but for more than half of all deaths on programme. However, it is also possible that deaths from tuberculosis may have been misattributed. In Agincourt a marked discrepancy was noted between physician and InterVA determinations for tuberculosis as a cause of death, particularly among older people, with a tendency for relative overattribution by the InterVA algorithm [15]. Tetanus is an extremely unlikely cause of death in our samples, since most cases worldwide are neonatal, and in the countries where we conducted our research the disease is largely controlled; the 1.7% of deaths attributed to this cause can therefore probably be discounted. Conversely, other causes of death are likely to have been underestimated. Cancer is thought to account for 13.1% of deaths among those aged 60 y and over in low income countries and 20.3% in middle income countries [37], yet only 2.3% of deaths were attributed to malignancy by InterVA. Dementia, a condition experienced by 450 of the 2,138 who died (21.0%), and known to be strongly independently associated with mortality risk [26],[38],[39], is not considered as a possible cause of death by the InterVA algorithm. These are examples of areas in which the algorithm could be adjusted to better reflect the relationship between symptoms and cause of death in varying contexts [29], in this instance, when applied to older people.

### Conclusions

The current global health agenda for chronic diseases is strongly premised on the concept of “premature mortality” and is informed, largely, by an attempt to reduce mortality among working age adults [40]. Our findings are important in informing priorities to improve health and reduce deaths in older people. They suggest an important latent independent protective effect of education even upon late-life mortality in countries with low and middle incomes. Given the much higher absolute mortality rates among older people, efforts to ensure universal access to education should confer substantial health benefits for generations to come. However, life course socioeconomic factors explain little of the variation in mortality rates between sites. This is consistent with Preston's classic observation that exogenous factors have contributed more to increases in life expectancy, particularly in poorer countries [41]; thus, interventions targeting social and economic vulnerability in late life and promoting access to effectively organised health care are also indicated [42]. Stroke, in particular, deserves much more attention. Millions of deaths in LMICs might be prevented using affordable and effective preventive strategies coupled with acute stroke management [30],[43]. Since strokes and deaths from stroke in these regions are concentrated among older people, it follows that these initiatives, and, indeed, chronic disease control programmes generally, need to be targeted accordingly.

## Supporting Information

Table S1 **Age distribution of the sample, by sex, in each site.**
(DOC)

Table S2 **Mortality rate (per 1,000 person-years) by age group, sex, and site.**
(DOC)

Table S3 **Predictors of loss to follow-up (vital status not identified).**
(DOC)

Table S4 **Predicted probability of death before follow-up compared between groups, according to vital status ascertainment.**
(DOC)

## Abbreviations

HR: hazard ratio
LMICs: low and middle income countries
SMR: standardized mortality ratio
VA: verbal autopsy
