# Supplementary material for: Socioeconomic Factors and All Cause and Cause-Specific Mortality among Older People in Latin America, India, and China: A Population-Based Cohort Study
Source: PLoS Med. 2012 Feb 28;9(2):e1001179. doi: 10.1371/journal.pmed.1001179 (PMC3289608; doi:10.1371/journal.pmed.1001179)
Supplement: Table S1 — Age distribution of the sample, by sex, in each site. (DOC) [file pmed.1001179.s001.doc]

Table S1: Age distribution of the sample, by sex, in each site

|  |  | 65-69  N (%) | 70-74  N (%) | 75-79  N (%) | 80-84  N (%) | 85-89  N (%) | 90+  N (%) |
| --- | --- | --- | --- | --- | --- | --- | --- |
| Cuba  (n=2637)  mv=7 | Female | 434 (25.4) | 437 (25.6) | 366 (21.4) | 255 (14.9) | 142 (8.3) | 76 (4.4) |
|  | Male | 237 (25.8) | 261 (28.4) | 211 (22.9) | 126 (13.7) | 61 (6.6) | 24 (2.6) |
|  | Total | 671 (25.5) | 698 (26.5) | 577 (21.9) | 381 (14.5) | 203 (7.7) | 100 (3.8) |
| Dominican Republic  (n=1706)  mv=2 | Female | 289 (25.6) | 275 (24.3) | 225 (19.9) | 171 (15.1) | 104 (9.2) | 66 (5.8) |
|  | Male | 153 (26.7) | 163 (28.4) | 121 (21.1) | 73 (12.7) | 44 (7.7) | 20 (3.5) |
|  | Total | 442 (25.9) | 438 (25.7) | 346 (20.3) | 244 (14.3) | 148 (8.7) | 86 (5.0) |
| Peru (urban)  (n=1245)  mv=1 | Female | 248 (30.8) | 198 (24.6) | 172 (21.4) | 100 (12.4) | 52 (6.5) | 34 (4.2) |
|  | Male | 96 (21.8) | 120 (27.3) | 96 (21.8) | 66 (15.0) | 48 (10.9) | 14 (3.2) |
|  | Total | 344 (27.6) | 318 (25.6) | 268 (21.5) | 166 (13.3) | 100 (8.0) | 48 (3.9) |
| Peru (rural)  (n=507)  mv=0 | Female | 96 (35.6) | 74 (27.4) | 47 (17.4) | 33 (12.2) | 17 (6.3) | 3 (1.1) |
|  | Male | 74 (31.2) | 54 (22.8) | 43 (18.1) | 34 (14.3) | 18 (7.6) | 14 (5.9) |
|  | Total | 170 (33.5) | 128 (25.2) | 90 (17.7) | 67 (13.2) | 35 (6.9) | 17 (3.3) |
| Venezuela  (n=1706)  mv=3 | Female | 461 (42.7) | 251 (23.3) | 187 (17.3) | 93 (8.6) | 54 (5.0) | 33 (3.1) |
|  | Male | 278 (44.5) | 160 (25.6) | 109 (17.5) | 53 (8.5) | 16 (2.6) | 8 (1.0) |
|  | Total | 739 (43.4) | 411 (24.1) | 296 (17.4) | 146 (8.6) | 70 (4.1) | 41 (2.4) |
| Mexico (urban)  (n=910)  mv=0 | Female | 171 (28.3) | 193 (31.9) | 111 (18.3) | 73 (12.1) | 45 (7.4) | 12 (2.0) |
|  | Male | 54 (17.7) | 107 (35.1) | 72 (23.6) | 48 (15.7) | 22 (7.2) | 2 (0.7) |
|  | Total | 225 (24.7) | 300 (33.0) | 183 (20.1) | 121 (13.3) | 67 (7.4) | 14 (1.5) |
| Mexico (rural)  (n=935)  mv=0 | Female | 186 (32.7) | 142 (25.0) | 128 (22.5) | 69 (12.1) | 34 (6.0) | 10 (2.0) |
|  | Male | 92 (25.1) | 96 (26.2) | 78 (21.3) | 68 (18.6) | 29 (8.0) | 3 (0.8) |
|  | Total | 278 (29.7) | 238 (25.4) | 206 (22.0) | 137 (14.6) | 63 (6.7) | 13 (1.4) |
| China (urban)  (n=980)  mv=0 | Female | 171 (30.5) | 164 (29.3) | 117 (20.9) | 65 (11.6) | 37 (6.6) | 6 (1.1) |
|  | Male | 95 (22.1) | 132 (30.8) | 105 (24.5) | 61 (14.2) | 31 (7.2) | 5 (1.1) |
|  | Total | 266 (26.9) | 296 (29.9) | 222 (22.4) | 126 (12.7) | 68 (6.9) | 11 (1.1) |
| China (rural)  (n=1002)  mv=0 | Female | 191 (34.3) | 165 (29.7) | 125 (22.5) | 46 (8.3) | 18 (3.2) | 11 (2.0) |
|  | Male | 192 (43.0) | 131 (29.7) | 77 (17.3) | 27 (6.0) | 17 (3.8) | 2 (0.5) |
|  | Total | 383 (38.2) | 296 (29.5) | 202 (20.2) | 73 (7.3) | 35 (3.5) | 13 (1.3) |
| India  (n=748)  mv=13 | Female | 178 (42.4) | 140 (33.3) | 54 (12.9) | 33 (8.0) | 10 (2.4) | 5 (1.2) |
|  | Male | 129 (40.9) | 88 (27.9) | 58 (18.4) | 18 (5.7) | 15 (4.8) | 7 (2.2) |
|  | Total | 307 (41.8) | 228 (31.0) | 112 (15.2) | 51 (6.9) | 25 (3.4) | 12 (1.6) |
