# Supplementary material for: Socioeconomic Factors and All Cause and Cause-Specific Mortality among Older People in Latin America, India, and China: A Population-Based Cohort Study
Source: PLoS Med. 2012 Feb 28;9(2):e1001179. doi: 10.1371/journal.pmed.1001179 (PMC3289608; doi:10.1371/journal.pmed.1001179)
Supplement: Table S2 — Mortality rate (per 1,000 person-years) by age group, sex, and site. (DOC) [file pmed.1001179.s002.doc]

Table S2: Mortality rate (per 1000 person years) by age group, sex, and site

| Site | Sex | **65-69 years**  **% (95%CI)** | **70-74 years**  **% (95%CI)** | **75-79 years**  **% (95%CI)** | **80+ years**  **% (95%CI)** | **All ages** |
| --- | --- | --- | --- | --- | --- | --- |
| Cuba | Female | 12.9 (7.5-20.8) | 26.3 (19.9-34.3) | 39.7 (21.1-50.0) | 101.9 (89.3-115.7) | 51.2 (46.1-56.6) |
|  | Male | 18.6 (10.1-31.7) | 34.4 (24.8-46.5) | 66.3 (51.2-84.4) | 133.7 (112.2-158.3) | 65.9 (58.0-74.5) |
| Dominican Republic | Female | 22.2 (13.8-34.0) | 28.0 (20.1-38.0) | 43.5 (32.6-52.9) | 102.4 (87.8-118.8) | 55.0 (48.7-61.8) |
|  | Male | 38.2 (23.0-59.9) | 57.6 (41.7-77.7) | 61.4 (44.1-83.4) | 143.4 (116.9-174.1) | 78.9 (68.3-90.6) |
| Peru (urban) | Female | 8.2 (3.0-18.2) | 7.0 (2.2-16.9) | 11.0 (4.5-22.9) | 53.4 (37.1-74.5) | 20.2 (15.0-26.8) |
|  | Male | 14.7 (3.7-40.1) | 20.6 (9.0-40.8) | 25.8 (12.0-49.1) | 80.7 (56.5-112.0) | 40.4 (30.4-52.7) |
| Peru (rural) | Female | 0 | 28.8 (11.3-51.1) | 21.8(6.9-52.5) | 49.0 (25.7-85.1) | 23.3 (14.9-34.6) |
|  | Male | 26.0 (9.5-57.7) | 4.6 (0.2-22.6) | 45.5 (19.9-89.9) | 74.9 (46.4-114.7) | 39.1 (27.2-54.5) |
| Venezuela | Female | 10.3 (6.0-16.7) | 11.0 (6.3-18.0) | 26.1 (16.8-38.8) | 58.8 (44.3-76.5) | 23.1 (19.0-27.9) |
|  | Male | 27.8 (18.2-40.7) | 30.5 (20.0-44.7) 53.65365 | 36.5 (22.3-57.6) | 67.3 (45.9-95.4) | 36.9 (30.0-44.9) |
| Mexico (urban) | Female | 6.9 (1.7-18.7) | 19.6 (10.3-34.2) | 23.4 (11.4-42.9) | 84.3 (59.6-115.9) | 32.3 (24.7-41.4) |
|  | Male | 43.6 (16.0-96.6) | 46.8 (26.0-78.0) | 24.8 (10.1-51.7) | 71.7 (43.2-112.5) | 47.1 (34.2-63.3) |
| Mexico (rural) | Female | 21.7 (11.0-38.7) | 20.5 (10.0-37.7) | 45.8 (27.6-71.8) | 65.3 (43.2-94.9) | 36.9 (28.5-47.1) |
|  | Male | 18.5 (5.9-44.6) | 35.5 (18.0-63.3) | 56.9 (30.8-96.7) | 70.4 (45.7-103.9) | 47.3 (35.4-62.0) |
| China (urban) | Female | 4.0 (0.7-13.1) | 21.7 (13.4-33.3) | 32.6 (20.7-49.0) | 100.0 (77.6-126.9) | 39.8 (32.7-47.9) |
|  | Male | 0.0 | 24.9 (14.7-39.6) | 54.7 (37.8-76.6) | 140.0 (110.2-175.6) | 60.0 (49.9-71.6) |
| China (rural) | Female | 5.4 (1.4-14.7) | 38.0 (26.7-52.5) | 78.8 (59.1-103.1) | 140.4 (109.0-178.2) | 59.4 (50.5-69.5) |
|  | Male | 12.1 (5.3-24.0) | 55.9 (40.8-74.8) | 91.6 (66.6-123.1) | 202.1 (151.8-264.6) | 69.2 (58.4-81.4) |
| India (urban) | Female | 31.5 (18.7-50.1) | 50.1 (32.2-74.5) | 71.6 (38.8-121.7) | 91.4 (52.0-149.7) | 50.5 (39.2-64.1) |
|  | Male | 50.4 (30.4-79.1) | 93.5 (61.9-136.0) | 108.5 (67.3-166.3) | 191.8 (123.2-285.6) | 92.8 (74.4-114.5) |
