# Supplementary material for: Socioeconomic Factors and All Cause and Cause-Specific Mortality among Older People in Latin America, India, and China: A Population-Based Cohort Study
Source: PLoS Med. 2012 Feb 28;9(2):e1001179. doi: 10.1371/journal.pmed.1001179 (PMC3289608; doi:10.1371/journal.pmed.1001179)
Supplement: Table S3 — Predictors of loss to follow-up (vital status not identified). (DOC) [file pmed.1001179.s003.doc]

Table S3: Predictors of loss to follow-up (vital status not identified)

| Site^1^ | Age group (per 5 year band) | Sex (Male vs. female) | Education (per level) | Disability  (WHODAS-12) | Dementia |
| --- | --- | --- | --- | --- | --- |
| Cuba | 1.00 (0.90-1.12) | 0.79 (0.58-1.09) | 1.15 (1.00-1.33) | 1.00 (0.99-1.01) | 1.17 (0.67-2.04) |
| Dominican Republic | 1.00 (0.92-1.07) | 1.06 (0.85-1.32) | 1.03 (0.92-1.15) | 1.00 (0.99-1.00) | 0.53 (0.33-0.86) |
| Peru (urban) | 1.05 (0.93-1.19) | 1.15 (0.82-1.61) | 0.94 (0.79-1.12) | 0.99 (0.98-1.00) | 1.54 (0.84-2.82) |
| Peru (rural) | 1.18 (0.97-1.43) | 0.90 (0.52-1.56) | 0.93 (0.68-1.28) | 0.99 (0.97-1.01) | 1.58 (0.53-4.75) |
| Venezuela | 1.09 (0.99-1.20) | 0.90 (0.70-1.15) | 0.94 (0.82-1.08) | 1.00 (0.99-1.01) | 1.03 (0.60-1.76) |
| Mexico (urban) | 0.98 (0.84-1.14) | 1.13 (0.75-1.69) | 1.04 (0.87-1.24) | 1.01 (0.99-1.02) | 1.89 (1.02-3.49) |
| Mexico (rural) | 1.10 (0.91-1.33) | 1.41 (0.88-2.24) | 1.20 (0.90-1.60) | 1.00 (0.98-1.01) | 0.47 (0.15-1.52) |
| China (urban) | 0.87 (0.77-0.97) | 0.84 (0.62-1.13) | 1.17 (1.04-1.31) | 1.01 (1.00-1.01) | 0.38 (0.15-0.96) |
| India | 0.99 (0.91-1.09) | 0.94 (0.75-1.19) | 1.00 (0.90-1.10) | 1.00 (0.99-1.01) | 1.15 (0.77-1.70) |

1. Vital status was ascertained for all participants in the rural China site
