# Supplementary material for: Socioeconomic Factors and All Cause and Cause-Specific Mortality among Older People in Latin America, India, and China: A Population-Based Cohort Study
Source: PLoS Med. 2012 Feb 28;9(2):e1001179. doi: 10.1371/journal.pmed.1001179 (PMC3289608; doi:10.1371/journal.pmed.1001179)
Supplement: Table S4 — Predicted probability of death before follow-up compared between groups, according to vital status ascertainment. (DOC) [file pmed.1001179.s004.doc]

Table S4 Predicted probability of death before follow-up compared between groups, according to vital status ascertainment

| Site^1^ | Vital status ascertained. Mean predicted probability of death | Vital status not ascertained. Mean predicted probability of death | Mean difference | t-value, p-value |
| --- | --- | --- | --- | --- |
| Cuba | 0.230 (0.004) | 0.222 (0.014) | -0.009 (-0.037 to +0.019) | -0.61, 0.54 |
| Dominican Republic | 0.271 (0.004) | 0.252 (0.008) | -0.019 (-0.039 to +0.001) | -1.89, 0.06 |
| Peru (urban) | 0.078 (0.003) | 0.077 (0.008) | -0.001 (-0.021 to + 0.018) | -0.15, 0.88 |
| Peru (rural) | 0.104 (0.005) | 0.112 (0.014) | +0.007 (-0.025 to +0.039) | 0.45, 0.66 |
| Venezuela | 0.110 (0,002) | 0.113 (0.006) | +0.003 (-0.010 to +0.016) | 0.44, 0.66 |
| Mexico (urban) | 0.110 (0.003) | 0.136 (0.014) | +0.027 (+0.004 to +0.049) | 2.31, 0.02 |
| Mexico (rural) | 0.118 (0.003) | 0.120 (0.013) | +0.002 (-0.021 to +0.026) | 0.21, 0.83 |
| China (urban) | 0.225 (0.007) | 0.172 (0.012) | -0.053 (-0.086 to -0.020) | -3.18, 0.002 |
| India | 0.199 (0.005) | 0.195 (0.010) | -0.004 (-0.025 to +0.016) | -0.40, 0.69 |

1. Vital status was ascertained for all participants in the rural China site
